# Age-associated DNA methylation changes in *Xenopus* frogs

**DOI:** 10.1080/15592294.2023.2201517

**Published:** 2023-04-24

**Authors:** Marco Morselli, Ronan Bennett, Nikko-Ideen Shaidani, Marko Horb, Leonid Peshkin, Matteo Pellegrini

**Affiliations:** a Molecular, Cell & Developmental Biology, UCLA, Los Angeles, CA, USA; b Eugene Bell Center for Regenerative Biology and Tissue Engineering and National Xenopus Resource, Marine Biological Laboratory, Woods Hole, MA, USA; c Systems Biology, Harvard Medical School, Boston, MA, USA

**Keywords:** Epigenetic clock, *Xenopus*, whole-genome bisulfite sequencing, DNA methylation, targeted bisulfite sequencing

## Abstract

Age-associated changes in DNA methylation have been characterized across various animals, but not yet in amphibians, which are of particular interest because they include widely studied model organisms. In this study, we present clear evidence that the aquatic vertebrate species *Xenopus tropicalis* displays patterns of age-associated changes in DNA methylation. We have generated whole-genome bisulfite sequencing (WGBS) profiles from skin samples of nine frogs representing young, mature, and old adults and characterized the gene- and chromosome-scale DNA methylation changes with age. Many of the methylation features and changes we observe are consistent with what is known in mammalian species, suggesting that the mechanism of age-related changes is conserved. Moreover, we selected a few thousand age-associated CpG sites to build an assay based on targeted DNA methylation analysis (TBSseq) to expand our findings in future studies involving larger cohorts of individuals. Preliminary results of a pilot TBSeq experiment recapitulate the findings obtained with WGBS setting the basis for the development of an epigenetic clock assay. The results of this study will allow us to leverage the unique resources available for *Xenopus* to study how DNA methylation relates to other hallmarks of ageing.

## Introduction

The quest for an accurate molecular measure of the age of an organism may eventually lead to the development of a better indicator of the health status and vulnerability to illnesses compared to chronological age. This could have profound implications for the management of health in humans, such as age-related disease prevention and improvement of quality of life. An ageing biomarker should be influenced by both genetic determinants and lifestyle factors, be easily and repeatedly (non-invasively) measured, and be easily transferable from model organisms to humans [1].

Many different ageing biomarkers have been developed, such as telomere length, proteins, metabolites, RNA levels, and epigenetic marks. Several studies have explored proteomic and metabolomic age predictors, but the vast majority relied on non-targeted approaches and/or were restricted to single cohorts, limiting their widespread use [2]. Other studies have examined telomere length, and the results show that while it can be used as a cell proliferation marker, it is only weakly correlated with age and mortality. The use of RNA levels as an ageing biomarker is attractive due to the ease of transcriptomic profiling. However, current ageing clocks based on transcriptomes show only modest accuracy and poor reproducibility. Recently, thanks to advancements in single-cell profiling, transcriptomic-based ageing biomarkers are also starting to be developed for single cells, enabling the measurement of ageing heterogeneity in tissues [3–7,8–10]. Despite the utility of these approachesto date, the most accurate molecular ageing predictors are based on epigenetic profiles. Many studies have examined chromatin accessibility and/or histone post-translational modifications to measure age [8,11], but DNA methylation shows much more robust changes with age [2,12]. Several studies have shown that DNA methylation is the most accurate marker of chronological age estimation, and ‘second generation’ clocks can also predict health span and mortality [13,14], bringing us a step closer towards a ‘biological age’ estimator [15]. The difference between the predicted age from these clocks and the actual age of an individual is commonly referred to as epigenetic age acceleration and has been shown to correlate with increased mortality risk [16]. Nonetheless, despite being extensively used as an ageing biomarker, the mechanisms underlying DNA methylation-based clocks are more difficult to interpret than transcriptomic and proteomic markers and cannot be applied to some of the model organisms widely used in ageing research, such as Caenorhabditis elegans, which lack DNA methylation.

Since the development of the first epigenetic clock in humans [17], DNA methylation age predictors have also been expanded to numerous other species, including mammals (e.g., rodents [18–23]), birds [24], reptiles [25], fish [26], and invertebrates [27], just to name a few. However, amphibians have not yet been extensively examined in ageing studies, despite the fact that *Xenopus* has many unique features as a model organism for ageing biology. *Xenopus* is thought to have negligible senescence [28,29], remains fertile late in life, provides an ease of surgical and biochemical manipulation of the embryos, permits genome perturbation and editing (morpholino, transgenics, TALENs, and CRISPR/Cas), and has large oocytes.

In addition, *Xenopus* is phylogenetically closer to humans compared to other aquatic organisms, has limbs, digits, lungs, a three-chambered heart, and approximately 80% of the identified human disease genes are present in *Xenopus* [30].

To date, *Xenopus* has not been widely used as an ageing model due to the lack of verifiably old animals and a relatively long lifespan (with an estimated maximum lifespan of over 30 y in *X. laevis* and 12 y in shorter lived *X. tropicalis*), even though some research into reproductive system ageing in amphibians has been carried out [28,29].

DNA methylation has been previously profiled in amphibians, both in *Xenopus laevis* and *tropicalis* [31,32].

Using WGBS (whole-genome bisulfite sequencing) previous studies have shown that CpG sites have an average global methylation level of 91% across different developmental stages in *X. tropicalis* [33], including gametes (sperms and spermatids) [34]. *Xenopus* genomes contain two DNA cytosine (C5) DNA methyltransferases (DNMTs): DNMT1 and DNMT3a, a maintenance and a *de novo* DNA methyltransferase, respectively [35,36] (Supplementary Figure S1). In mammals, in addition to the previously mentioned DNMTs, there is an additional *de novo* enzyme (DNMT3b), an inactive DNMT-like accessory factor (DNMT3L), and a rodent-specific enzyme involved in male germline retrotransposon silencing (DNMT3c) [37,38]. The majority of mechanistic studies on DNA methylation in metazoans have been performed in human and mouse where they have found that de novo DNA methylation is deposited by two complexes (DNMT3a-DNMT3L mainly in intergenic regions and DNMT3b-DNMT3L mainly in intragenic regions), while its maintenance is governed by DNMT1 together with its accessory factor UHRF1, both localized to replicating foci. One of the roles of DNA methylation is to reinforce the repression of transposable elements [37,39,40].

Previous reports have shown that DNA methylation and histone modifications are highly interconnected in *X. tropicalis*, and similarly to mammals, 5^me^C levels are anticorrelated with H3K4me3 and H3K27me3 marks [31,32,41], with many promoters being hypomethylated [42]. Moreover, H3K36 methylation is important for the recruitment of DNMT3s through their PWWP domain [43–46]. Many other factors, such as DNA demethylases, methyl DNA binding proteins, and PTMs of DNMTs, in addition to DNA sequence and its accessibility, determine the DNA methylation landscape [47]. The similarity of patterns of DNA methylation in *X. tropicalis* and mammals suggest it is a highly conserved mechanism, despite an apparent absence of DNMT3b and the inactive accessory protein DNMT3L in *X. tropicalis*.

The goal of this study is to identify age-associated DNA methylation changes in *Xenopus tropicalis* and to lay the groundwork for the development of an efficient epigenetic clock. To this end, we leveraged samples from the National Xenopus Resource at the Marine Biology Laboratory that has been maintaining inbred *Xenopus* animals for over 10 y creating a unique collection of animals of known age, covering ages from 1 to 10 y. This resource allows the establishment of a cohort covering a wide range of confidently determined ages. We collected samples from skin tissue thanks to established protocols for sampling without sacrificing the animal, allowing longitudinal studies. We identified age-associated 5^me^CpG sites through WGBS of nine frogs (three different age groups) and validated a targeted bisulfite sequencing (TBSeq) assay in a pilot experiment with sixteen frogs (nine different age groups). This sets the basis for future interrogation of DNA methylation in larger cohorts of frogs and the development of a robust epigenetic clock.

## Results and discussion

In order to assess age-related changes in DNA methylation, we chose to focus on three ages, covering the range from young to old adults. The National Xenopus Resource has maintained animal stocks for 10 y and therefore some of these animals represent some of the oldest available individuals known to the Worldwide *Xenopus* research community. The youngest animals represent adults which have been reproducing for a few months as sexual maturity in *X. tropicalis* comes at 4–6 months depending on husbandry conditions [48,49].

**Figure 1.**
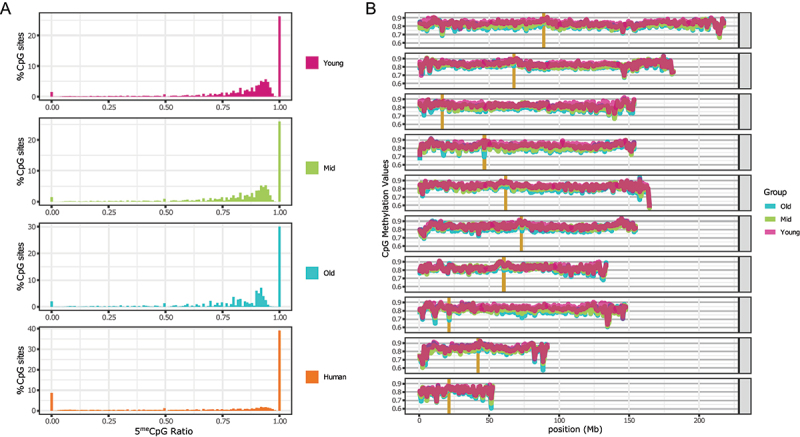
Cytosine methylation levels in CpG context. (A) Distribution for *X. tropicalis* of three different age groups and human mammary epithelial tissue. For each *X. tropicalis* age group the average of three different individuals. Common sites with at least 5× coverage were used. The human mammary epithelial dataset is from ENCODE (doi:10.17989/ENCSR656TQD, file ENCFF699GKH). (B) CpG methylation distribution over chromosomes for each age group (average of all samples within the same group). Vertical gold bars represent centromere positions. Common sites for all samples with at least 3× coverage were used. Resolution 500Kb.

The samples were collected from the skin of the webbing in the hindlimb of nine individuals of *Xenopus tropicalis*. Cytosine DNA methylation was measured by WGBS with a sequencing depth ranging between 9× and 15× (see Materials and Methods, Supplementary Table S1). The resulting methylation showed a distribution typical of the methylation of higher eukaryotes (Figure 1a), with the majority of the CpG sites fully methylated or highly methylated (approximately 70% of the CpG sites show >80% methylation) and only a small fraction completely unmethylated or slightly methylated (approximately 3% of the CpG sites show <5% methylation). A similar pattern is observed in mammals as exemplified by the human mammary epithelial tissue (Figure 1a). DNA methylation in other contexts (CpHpH and CpHpG) was low (Table 1). The small differences in the patterns between frog and human samples can be partially attributed to the differences in tissue purity of the cell-type composition between the epithelial samples in human and skin punches in frog.

**Table 1.** Average 5^me^C (%) by dinucleotide context.

Sample	Age (years)	Strain	CpG	CpA	CpC	CpT
Young_1	1	wt nigerian	82.19	00.93	00.54	00.59
Young_2	1	wt nigerian	82.49	00.93	00.56	00.61
Young_3	1	wt nigerian	82.31	00.84	00.51	00.53
Mid_1	5	no privacy	80.25	00.83	00.48	00.50
Mid_2	5	eef1a1:GFP	81.31	00.83	00.50	00.53
Mid_3	5	eef1a1:GFP	80.19	00.86	00.53	00.55
Old_1	9	eef1a1:GFP	79.17	01.38	00.74	00.95
Old_2	9	eef1a1:GFP	80.11	00.99	00.56	00.62
Old_3	9	eef1a1:GFP	80.36	00.93	00.54	00.58

Taking advantage of recently published high-quality chromosome-level assembly of the *X. tropicalis* genome, we examined chromosome-level DNA methylation patterns (Figure 1b). The CpG methylation levels over 500 Kb-bins are high across the genome with only a few dips towards the ends of a few chromosomes (e.g., subtelomeric regions of Chr5 and Chr9) but never below 60%. At this level of signal smoothing, there is a noticeable difference in how uniform the levels of DNA methylation are across the chromosomes – towards the telomeric regions, and there is much higher variability in every chromosome. This spatial pattern is conserved across all three ages. Since peri-centromeric chromatin is known to be highly repetitive, and DNA methylation patterns are known to decorate genomic repeats, we noted the centromeres of the *X. tropicalis* chromosomes in Figure 1b. The centromeric regions of most of the chromosomes (Chr 2, 4, 6, 7, 8 and 9) appear to be hypo-methylated similarly to the human DNA [50].

**Figure 2.**
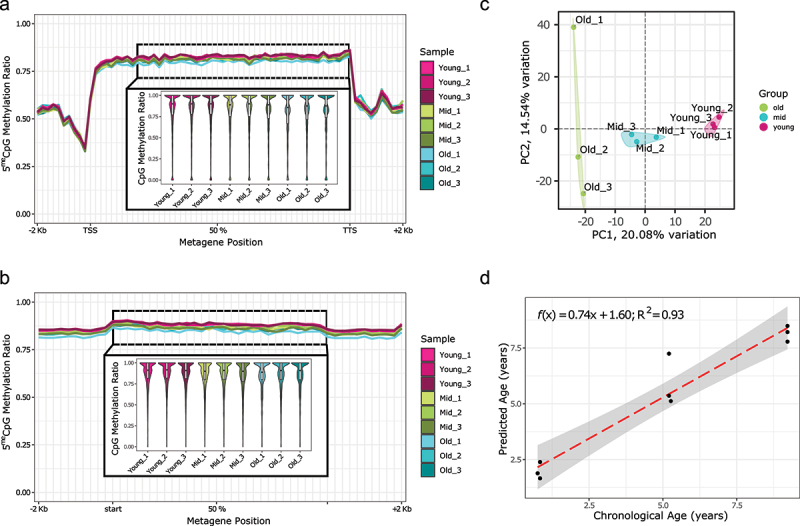
CpG methylation distribution over genomic features. (A) Gene metaplot with upstream and downstream 2Kb. In the inset, a zoom in of the central part of the gene body (from the second decile to the TTS). (B) Gene body CpG methylation in repeated elements bigger than 1Kb [not distinguished by class nor family]. (C) Principal Component Analysis (showing PC1 vs. PC2) using CpG methylation levels for the nine frog samples. CpG sites with at least 5× coverage were used and filtered to remove the variables with low variance (PCAtools, removeVar = 0.1). (D) CpG methylation preliminary clock leave-one-out predictions. Each point represents the age prediction on one sample from an elastic net model which was trained on the other eight samples. For all panels, common sites for all samples with at least 5× coverage were used. The boxplots in the insets show the mean of the distribution.

Consistent with what we know from mammalian DNA methylation patterns, the global methylation levels of CpG sites are slightly but statistically significantly different among the three age groups (based on a pairwise two-sample Kolmogorov–Smirnov test on the age group-combined common CpG sites covered by at least ten reads). The highest levels are seen in the ‘young’ group (approx. 1 y/o), intermediate levels are found in ‘Mid’ (approx. 5 y/o), and the lowest levels are observed in the ‘Old’ group (approx. 9 y/o) (Table 1, Supplementary Table S1). The same pattern of methylation reduction with age is seen in both genes (Figure 2a) and repeated elements (Figure 2b). Similarly, to mammalian 5^me^C distribution, the gene body is highly methylated (average methylation levels are above 75%) and the region within 1Kb of the TSS has lower levels of DNA methylation, as previously reported [42]. This is likely due to the presence of histone marks that repress DNA methylation (e.g., H3K4 methylation) in the promoters of expressed genes, or other histone-based repressive marks (e.g., H3K27 methylation) in the promoters of non-expressed genes. Conversely, repeated elements (longer than 1 Kb) show high methylation levels (average >80%) spanning their entire length. The methylation levels of common CpG sites covered by at least five reads can also be used to discriminate the samples of the three age groups through a principal component analysis (Figure 2c). Principal Component 1 (PC1) has a high correlation with age (R^2^ = 0.98), while other PCs show no correlation (Supplementary Figure S2). Here again, consistent with mammalian age-related methylation patterns, we see lower variance across young samples compared to old samples with middle age samples showing intermediate variability.

We also performed an autocorrelation analysis based on the CpG methylation level of the 20 closest CpG sites (Supplementary Figure S3). This analysis shows an expected decrease in the correlation of CpG methylation levels as the relative CpG position increases, but it highlights how young samples have higher correlations than samples with an intermediate age. The old samples show the lowest autocorrelation values. This might be partially explained by a progressive loss of DNA methylation maintenance activity (through DNMT1-UHRF1, or other recruiting factors) [8,51].

We next asked whether we can use the CpG methylation levels to predict the age of an individual. Normally, a much larger number of samples would be required to build and validate a methylation-based ageing model, but three ages with three repeats each allow us to construct a preliminary proof-of-principle model. We used elastic net regularized linear regression for the model. The predictors were the methylation values at CpG sites across the genome with 5× coverage in all samples (110544 sites, the same used in Figure 2c). By performing leave-one-out cross validation (LOOCV), we achieved a significant correlation between actual age and predicted age (R^2^ = 0.93) (Figure 2d) and a MAE = 1.0017 y. However, the age predictions of the young frogs are overestimated, and underestimated for old frogs. The LOOCV procedure involved fitting nine elastic net clock models, which altogether selected 331 distinct CpG sites as relevant features (Supplementary Figure 4, Supplementary Table 2).

**Figure 3.**
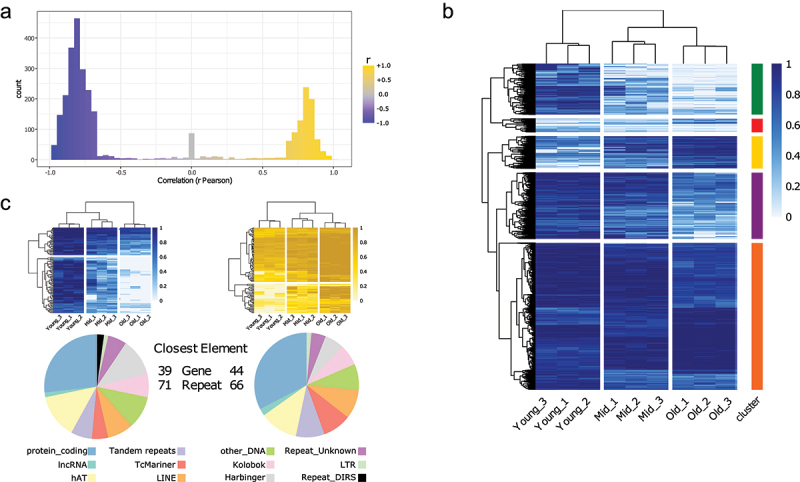
Selection of CpG sites correlated with age. (A) Pearson correlation (r) distribution of the approximately 3400 CpG sites selected. (B) CpG methylation levels of the selected sites (same as in A). Sites are grouped in five clusters, based on the clustering (method = ‘complete’). (C) CpG methylation levels of the top 100 sites correlated with age (right, gold), and anti-correlated (left, blue) (top). Distribution of the closest genomic elements (both genes and repeats) for each site (bottom).

In order to expand and confirm the findings in a bigger cohort, while avoiding the high costs of WGBS, we selected a few thousand sites (within approximately 3400 regions) that are strongly (both positively and negatively) correlated with age (Figure 3a, Supplementary Table 3). Using the CpG methylation levels of the selected sites, we were able to cluster nine samples based on age groups (Figure 3b). As expected, the majority of the sites show a noticeable decrease in DNA methylation levels from young to old samples (green and purple clusters), but a fraction (approx. 10% – gold cluster) shows an inverse pattern. Overall, we observe hundreds of highly informative sites with different age associated patterns. Some sites are modified between young and middle ages while others between middle and old. It is clear that while the overall similarity of the DNA methylation patterns is diminishing with age, the most informative sites are most discordant in the middle age, suggesting that the trajectories from young to old diverge in the middle age. As expected, the top 100 anticorrelated sites show a global decrease in methylation as samples increase with age (Figure 3c, top left, blue heatmap) and they are mostly found within repetitive elements (Figure 3c, bottom left, pie chart). Conversely, the top 100 correlated sites show rising DNA methylation levels as sample age increases (Figure 3c, top right, gold heatmap) with a similar trend in associated genome elements (Figure 3c, bottom right, pie chart).

To gain additional insights into the biological relevance of the age-associated CpG sites, we first linked each site to the nearest genomic element (combined genes and repeats annotation) (Supplementary Table 4). The selected regions are mainly found within genes and repeated elements, and less than 20% of them can be found outside of the elements, but always within 3 Kb (Supplementary Figure 5). We performed gene enrichment analysis with the PANTHER Overrepresentation Test using the list of site-linked genes (disregarding the repeats) against a reference with all *Xenopus tropicalis* genes. We interrogated different databases (Supplementary Table 5) and the enriched terms are mainly involved in cell-to-cell contacts (e.g., cell–cell adhesion GO:0098609, FDR = 1.31E–08;) and communication, mainly neuron-related (e.g., modulation of chemical synaptic transmission GO:0050804, FDR = 6.92E–06;), and, albeit with a lower FDR, intracellular signalling (e.g., guanyl-nucleotide exchange factor activity GO:0005085, FDR = 1.49E–03; adenylate cyclase-activating G protein-coupled receptor signalling pathway GO:0007189, FDR = 3.17E–02; regulation of protein kinase B signalling (GO:0051896), FDR = 4.48E–02). A plausible interpretation of these results might be that with age cells of the skin tissue, such as keratinocytes, might have an altered ability to communicate with and respond to other cells, a well-known hallmark of ageing [52].

Interestingly, the term ATP-dependent DNA helicases (blm, recql5, mcm8, chd5, wrn, smarca2, chd8) is also significantly enriched (GO:0008094, FDR = 3.78E–02) and includes enzymes not only involved in DNA replication and gene regulation but also in DNA repair, another one of the hallmarks of ageing [53]. We also see under-represented terms, such as reverse transcriptase (PANTHER protein class PC00200, FDR = 7.55E–04), most likely an artefact for the removal of repeat-linked terms during the creation of the query list, and C2H2 zinc finger transcription factor (PC00248, FDR = 3.04E–02), mainly repressive (klf7, klf8, sall1, znf407, znf462, znf536, bcl11a, prdm16).

The majority of the sites identified as predictive in the LOOCV preliminary clock are also found within the regions correlated with age (255 out of 331 predictive sites, 77%) (Supplementary Figure 6A). Among the 76 predictive CpG sites that are not located within the correlated regions, the vast majority (61, approx. 80%) are only employed in 1 model, and their methylation levels are not sufficient to cluster the samples based on their age (Supplementary Figure 6B). Conversely, the predictive sites within the regions can cluster the samples based on age and two main types of CpG sites can be observed: sites where the CpG methylation level increases with age (top, approx. 16%) and sites showing a decrease in methylation with age (bottom, approx. 84%) (Supplementary Figure 6C).

To test whether our findings could be extended to a different cohort of samples, we synthesized biotinylated DNA probes complementary to our age-associated sites and performed Targeted Bisulfite Sequencing (TBSeq), an assay that assesses the 5^me^C levels in a small fraction of the genome with high coverage [54]. In this pilot experiment, we interrogated approximately 3500 regions in the DNA extracted from hindlimb webbing of 16 different *X. tropicalis* frogs with the following ages: 2.3, 3.6, 4.2, 4.6, 6.9, 7.9, 8.5, 8.7, 10.9 y (Supplementary Table 6). Preliminary results show that the assay performs well (high coverage on the targeted regions, good ON/OFF target ratio) and we were able to generate a methylation matrix with more than 27’000 CpG sites (common to all samples and covered by more than 100 reads). We performed principal component analysis, which revealed that the combination of PC1 and PC2 is sufficient to discriminate the age groups: while PC1 mainly segregates the old group (>10 y old) from the young and mature groups (ranging between 2 and 8 y), PC2 seems to differentiate samples within the young and mature groups (Supplementary Figure 7A). A correlation analysis (Spearman) using the same CpG sites (common, 100× coverage) followed by hierarchical clustering shows similarities within the various age groups (Supplementary Figure 7B). Clustering of the top 500 variable 5^me^CpG sites illustrates age-dependent DNA methylation patterns (Supplementary Figure 7C), with the majority of the sites showing hypomethylation in older samples only and intermediate and hypermethylation in the remaining samples (three leftmost clusters), while only a small fraction shows hypermethylation in the old samples with low to intermediate methylation in other samples and an even smaller fraction (rightmost cluster) showing age-independent variable methylation. These results demonstrate that our assay based on capture hybridization followed by bisulfite conversion and next-generation sequencing can differentiate samples of different ages.

## Limitations of the study and future directions

Our goal is to create a robust assay to investigate ageing biology in *X. tropicalis* clock, a widely studied model amphibian. Our study has a few limitations. First, it is based on the methylation patterns of only nine frogs. Moreover, despite the fact that the ages of the frogs range between 1 and 9 y, they are grouped into only three classes (1, 5, and 9 y). Therefore, our age prediction model is simply a proof of principle that cannot be generalized to other studies. The construction of an epigenetic clock requires substantially more samples in order to demonstrate the robustness of the model [55]. As a first step towards this goal, we used TBSeq in a pilot experiment to determine whether our selected age-associated sites could recapitulate our findings in a different cohort. Our results suggest that this method can be used to build an epigenetic clock in larger cohort.

Another limitation of our study is that we have not attributed specific methylation changes to specific cell types, even though a webbing sample contains not only skin but also connective tissue and blood. Nonetheless, we expect that future methylation clocks will be applicable to distinct frog tissues just as they are in mammals.

*Xenopus* is an important model organism for the study of embryology. Since the embryo develops outside of the mother’s organism, it is easily accessible for manipulation and observation, including embryonic grafts from transgenic animal lines. The present work, however, does not examine development in frogs, and we therefore leave this direction for future studies. Instead, this work is based on skin samples which are easily available and do minimal harm to the animals, with repeated sampling possible after animals are given a few months break, which allows one to study longitudinal changes in one animal. This in turn enables new directions for studying the causality of methylation and tracking the establishment of methylation patterns for the sites correlated with age across cell types. In future studies, it will be interesting to co-profile other molecules, such as RNA and/or protein (even at the single-cell level), in addition to tissue cellular composition to generate further insights into the mechanisms of ageing and correlated changes with our selected 5^me^C sites.

## Materials and methods

### Sample collection

Nine adult *Xenopus tropicalis* were housed at the National Xenopus Resource (RRID:SCR_013731) in two multi-rack recirculating aquatic systems with established diet and water parameters (conductivity, pH, and temperature) as previously defined [48,49]. Frogs were kept in tanks with constant temperature of 25°C and 12/12-h light cycle.

Disposable biopsy punches were used to collect tissue (VWR 21,909–140) from the hindlimb webbing of mutant (no privacy), transgenic (eef1a1:GFP), and wild type *X. tropicalis* with Nigerian St.549 background (RRID_NXR_1018; https://www.xenbase.org/entry/stockCenter/showLine.do?method=displayLine&lineId = 1146&). Both non-wt strains are not expected to cause differences in DNA methylation values in the sampled tissue. For the ‘no privacy’ heterozygous mutant (Xtr.hps6n^Grngr^; RRID:NXR_10019), the causative mutation has been identified in the homolog of the human Hermansky-Pudlak Syndrome 6 (HPS6) gene, part of the BLOC-2 complex (biogenesis of lysosomal organelles complex 2) that causes a pigmentation defect [56] (https://www.xenbase.org/entry/stockCenter/showLine.do?method=displayLine&lineId = 1234&). eef1a1:GFP is a transgenic strain (Xtr.Tg(eef1a1:GFP)^Krieg^; RRID:NXR_1008) expressing GFP from the promoter of ef1a (elongation factor 1-alpha) [57,58] (https://www.xenbase.org/entry/stockCenter/showTransgene.do?method=displayTransgene&transgeneId = 19777942&).

### DNA extraction

DNA extraction was performed from web punches (4 mm in diameter except 6 months old for which we did 2 mm), according to Xu Y. et al. [59] with minor modifications. Briefly, tissue chunks were digested o/n in a 1.5 ml tube containing 200 µl of lysis buffer (100 mM Tris-HCl pH 8; 200 mM NaCl; 0.20% SDS; 5 mM EDTA) + 4 µl of Proteinase K (20 mg/ml, NEB) at 55°C (300 rpm continuous shaking in a thermomixer). After centrifugation for 15 min at 16,000 g (room temperature), the supernatant was transferred into a new tube avoiding the debris. The centrifugation step was repeated after the addition and mixing of the same volume of isopropanol. The resulting pellet was then washed twice with 500 µl of EtOH 70% (centrifugation at 16,000 g for 10 min, RT). The remaining ethanol was then carefully removed and the pellet air dried for 5–10 min at 55°C (open caps). The dried pellet was then resuspended with 55 µl of EB buffer (10 mM Tris-HCl pH 8) at 55°C for 1 h at 1400 rpm in a thermomixer, before being quantified (Qubit dsDNA BR – LifeTechnologies) and quality checked (Agilent 4200 TapeStation – Genomic Assay).

### WGBS library preparation

One microgram of purified DNA was sonicated using the Bioruptor Pico (Diagenode) for 15 cycles of 30-s ON/90 s OFF. NEB Next Ultra II DNA kit was used for end-repair, A-tailing, and ligation of pre-methylated unique-dual indexed adapters [54]. Bisulfite conversion was performed with EZ DNA Methylation-Gold (Zymo Research) according to the manufacturer’s instructions. The final amplification was performed with KAPA HiFi U+ (Roche Sequencing), IDT xGen Primers (20 µM – Integrated DNA Technologies), for a total of 12 PCR cycles.

Library QC was performed using the D1000 Assay on a 4200 Agilent TapeStation, and its concentration measured with the Qubit dsDNA BR Assay (LifeTechnologies). Libraries were sequenced on a NovaSeq6000 (S4 lane) as paired-end 150 bases.

### TBSeq (targeted bisulfite sequencing) library preparation

Five hundred nanograms of purified DNA was sonicated using the Bioruptor Pico (Diagenode) for 15 cycles of 30-s ON/90 s OFF. TBSeq libraries were prepared as previously described [60] except that biotinylated DNA probes (synthesized by IDT Technologies) complementary to the regions of interest of the *Xenopus tropicalis* genome were used in the hybridization capture. Library QC was performed as described above for the WGBS libraries. The final pool was sequenced on a NovaSeq6000 (SP lane) as paired-end 150 bases.

### Data processing

Demultiplexed Fastq files were subject to QC (FastQC – Babraham Bioinformatics) and trimming with cutadapt v2.10 [61] (options: -u −10 -U 10 -q 20 -m 50) before alignment to the *Xenopus tropicalis* genome (version XENTR_10.0) with BSBolt Align [62] (default options). PCR duplicates were removed with samtools markdup (option -r) v1.15 [63]. DNA methylation was called using BSBolt CallMethylation v1.3.0 (options: -BQ 10 -MQ 20 -IO) resulting in CGmap files. The matrices of common CpG sites (with at least 3× or 5× coverage) were produced using BSBolt AggregateMatrix. For the combined CGmap files, deduplicated bam files from each sample were merged into one bam file per age group (YOUNG, MID, OLD). DNA methylation was called as described above and the CpG matrix computed with all the common sites covered by a predefined threshold (3× used in Figure 1 B; 5× used in Figure 1a; 10× used for statistical analysis).

Data for metagene plots were calculated with CGmap tools (tools: bed2fragreg; mfg) [64]. Data for chromosome-wide DNA methylation distribution were calculated with CGmap tools (tool: mbin).

Average coverage for the TBSeq data was calculated using the mosdepth tool [65] with the bed file listing the target regions and each sample bam file. The CGmatrix of the TBSeq samples is comprised of common CpG sites with a coverage higher than 100 × .

Data from human mammary epithelial tissues were downloaded from the Encode database (doi:10.17989/ENCSR656TQD, file ENCFF699GKH).

All plots were generated in R (version 4.1.2). PCA was performed with the PCAtool package (v2.6.0) in R [66]. The preliminary methylation clock model was created with the ElasticNet function from the Python module sklearn.linear_model [67]. The chosen hyperparameters (alpha = 0.00283693 and l1_ratio = 0.5) were found via grid search with a LOOCV procedure with the ElasticNetCV function (code available at the repository: https://github.com/ronanbennett/xenopus-aging). The correlation and ANOVA analyses to select sites associated with the three different age groups were performed using the cor.test (Pearson) and anova functions in R, respectively. The top 4500 sites (ranked by correlation and ANOVA adjusted p-values) were selected for probe design, resulting in a final synthesis capturing approximately 3400 sites of the initial list. The closest genomic element for each site was calculated using bedtools closest tool (−t all option) using a combined annotation with repeats (from Xentr10.repeatMasked.gff) and genes (from XENTR_10.0_Xenbase.gff3). GO Enrichment was performed with PANTHER (PANTHER Overrepresentation Test – Released 20,221,013, via http://geneontology.org/) using the genes associated with the probes (discarding the repeats) as a test list (approx. 960 uniquely mapped IDs out of approx. 1400 uploaded) and all the *Xenopus tropicalis* genes as a reference list (total of 22,504 IDs, PANTHER version 17.0 Released 2022-02-22).

## Supplementary Material

Supplemental Material

## Data Availability

Data are publicly available at the NCBI Gene Expression Omnibus SuperSeries GSE222108 (https://www.ncbi.nlm.nih.gov/geo/). The SuperSeries is composed of two SubSeries: GSE221656 for WGBS and GSE222107 for TBSeq.
