# Supplementary material for: Age-associated DNA methylation changes in Xenopus frogs
Source: Epigenetics. 2023 Apr 24;18(1):2201517. doi: 10.1080/15592294.2023.2201517 (PMC10128463; doi:10.1080/15592294.2023.2201517)
Supplement: Supplemental Material [file KEPI_A_2201517_SM4103.zip › Supplementary files/Supplementary Materials caption.docx]

# Supplementary Materials

**Supplementary Table 1:** WGBS sequencing stats

**Supplementary Table 2:** Predictive CpG sites coordinates.

**Supplementary Table 3:** Selected regions (probes) coordinates.

**Supplementary Table 4:** Selected regions (probes) nearest genomic elements (repeats/gene).

**Supplementary Table 5:** PANTHER GO analysis. Databases interrogated: PANTHER Protein Class; PANTHER GO-Slim Biological Process, Cellular Component, and Molecular Function. For GO-Slim results, only the most specific subclass is shown (parent terms removed). The terms are sorted by FDR, and only terms with an FDR <0.05 are shown.

**Supplementary Table 6:** TBSeq samples and stats.

**Supplementary Figure 1:** Comparison of DNA methyltransferases and main accessory proteins from *H. sapiens* (*Hs*) and *X. tropicalis* (*Xt*).

(A) *De novo* DNA methyltransferases: XtDNMT3a (accession number F7BU15), HsDNMT3a (Q9Y6K1), HsDNMT3b (Q9UBC3), and HsDNMT3L (Q9UJW3). PWWP domain: Pro-Trp-Trp-Pro motif (fuchsia) ; ADD domain: ATRX-DNMT3-DNMT3L, comprised of an N-terminal C2C2-type zinc finger (GATA-like), an imperfect PHD-type C4C4 zinc finger PHD finger and a C-terminal α-helix (olive). DNA-(cytosine-C5)-Methyltransferase Domain: catalytic domain (light blue). DNA-(cytosine-C5)-Methyltransferase Domain – like: inactive methyltransferase domain in DNMT3L (white with light blue zig-zags).

(B) Maintenance DNA methyltransferases: XtDNMT1 (F6QE78); HsDNMT1 (P26358); XtUHRF1 (F6UA42); HsUHRF1 (Q96T88). DMAP1-BD: interaction module that binds the transcriptional co-repressor DMAP1 (DNA methyltransferase-associated protein 1) (purple). PCNA-BD: Proliferating cell nuclear antigen interaction domain (lavender). In XtDNMT31, the PCNA-BD is colored with lavender stripes because no domain was predicted in this region despite the high levels of sequence identity in this region. NLS: nuclear localization signal (green). No prediction present for XtDNMT1. DRFtD: DNA replication foci-targeting domain (salmon). Despite the sequence similarity, the predicted domain in XtDNMT1 is shorter than the human counterpart (salmon diagonal stripes in the missing regions). CXXC-type: CXXC zinc finger domain (dark grey). Autoinhibitory linker, not predicted in XtDNMT1 despite high sequence similarity (red/diagonal red stripes). BAH1/BAH2: Bromo-adjacent homology domain (brown/dark orange). (GK)n: glycine-lysine (GK) repeats (bright yellow). DNA-(cytosine-C5)-Methyltransferase Domain: catalytic domain (light blue). Ub-like: ubiquitin-like domain (blue). TTD: tandem Tudor domain (orange). PHD: Plant Homeo Domain finger (grey). SRA: SET and RING-finger Associated domain containing the YDG motif, 5^me^C binding pocket, and residues important for base flipping (gold). RING: RING (really interesting new gene) zinc-finger (light green).

Diagonal black-striped boxes indicate a predicted disordered consensus sequence. Domains in the Xt proteins have been compiled from InterProScan predictions.

**Supplementary Figure 2:** Predictive CpG sites. (A) Number of CpG sites used in N models. E.g. The CpG sites in the category “Number of models = 9” correspond to the sites employed by all 9 models; the CpG sites in the category “Number of models = 5” correspond to the sites employed in 5 models only (9 models total). (B) Distribution of the closest genomic elements for all CpG sites used in at least one model (left, corresponding to all the 331 CpG sites) and for the CpG sites common to all 9 models (right, corresponding to the black bar in Supplementary Figure 2A, for a total of 57 CpG sites). Refer to Supplementary Table 2 for the list of the predictive CpG sites.

**Supplementary Figure 3:** (A) Distribution of distances between selected regions (refer to Supplementary Table 3) and genome elements: genes (left) and repeats (right). (B) Distribution of the closest genomic elements (both genes and repeats) for each selected region.

**Supplementary Figure 4:** (A) Overlap between the 331 predictive CpG sites (divided by the number of models they appear in) and the 3441 selected regions (light blue). (B) DNA methylation levels of the predictive CpG sites not overlapping with the selected regions (green gradient). (C) DNA methylation levels of the predictive CpG sites overlapping with the selected regions (purple gradient).

**Supplementary Figure 5:** (A) Correlation (Pearson) between samples’ age and Principal Components (PC), p-value and adjusted p-value (Holm method). (B) Scatterplots between PCs and samples’ age.

**Supplementary Figure 6:** Autocorrelation of CpG DNA methylation for the closest 20 CpG sites (Lag). Lag =0 (the site itself) has been omitted for all samples (r =1).

**Supplementary Figure 7:** Pilot Targeted Bisulfite Sequencing (TBSeq) on 16 *X. tropicalis* samples using the selected age-associated CpG sites. (A) Principal component analysis using common CpG sites covered by at least 100 reads filtered to remove the variables with low variance (PCAtools, removeVar = 0.1). Samples are colored based on age. (B) Pearson correlation followed by hierarchical clustering using all 100x covered common CpG sites. (C) Heatmap showing the DNA methylation levels of the most variable CpG sites (approximately 500 sites).
